# Supplementary material for: High-throughput genetic manipulation of multicellular organisms using a machine-vision guided embryonic microinjection robot
Source: Genetics. 2024 Feb 19;226(4):iyae025. doi: 10.1093/genetics/iyae025 (PMC10990426; doi:10.1093/genetics/iyae025)
Supplement: iyae025_Supplementary_Data [file iyae025_supplementary_data.zip › Supplemental_Figures_GENETICS-2023-306540.pdf]

SUPPLEMENTARY FIGURES

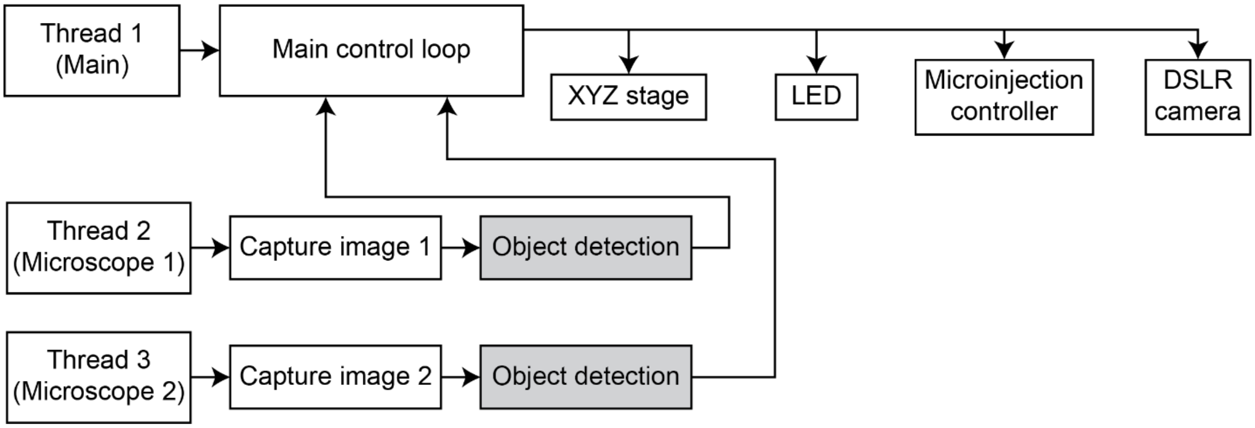

Figure S1: Software architecture for robot: Overall software architecture used for automated microinjection of zebrafish and Drosophila embryos.

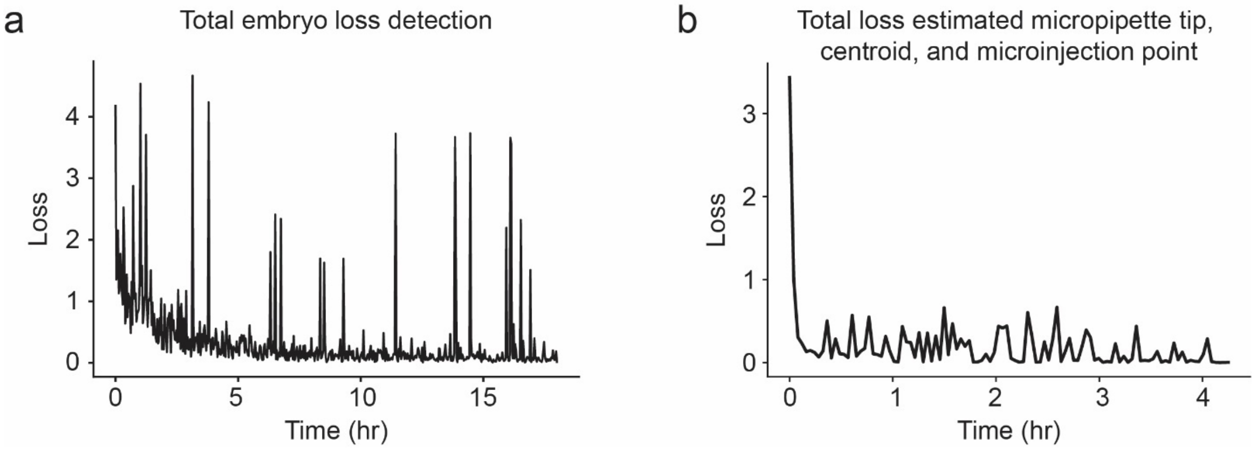

Figure S2: Machine learning loss rate for Drosophila dataset training: (a) ML loss rate for the model used to detect embryos at macroscale via the DSLR image. (b) ML loss rate for the model used to detect the micropipette tip, centroid of the embryo, and microinjection point of the embryo.

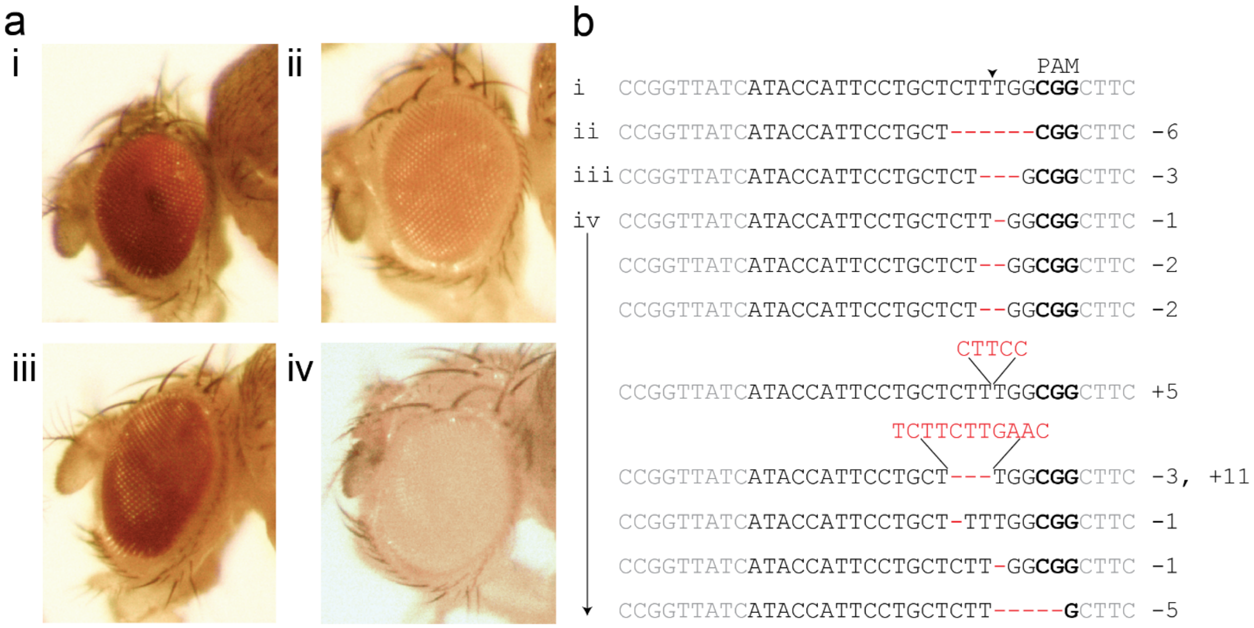

Figure S3: Molecular characterization of CRISPR *white* mutations: Mutations obtained via CRISPR mutagenesis. (a, I and b, i) Wild-type fly and DNA sequence, (a, ii and b, ii) hypomorphic mutant fly with a 6 bp (non-frameshifting) deletion, (a, iii and b, iii) hypomorphic mutant fly with 3 bp (non-frameshifting) deletion, and (a, iv and b, iv) example image of a null mutant fly and different classes of mutations detected with null mutant phenotypes, including a frameshifting 5 bp insertion, frameshifting 1 bp, 2 bp, and 5 bp deletions, and a complex frameshifting allele carrying a 3 bp deletion coupled with an 11 bp insertion.

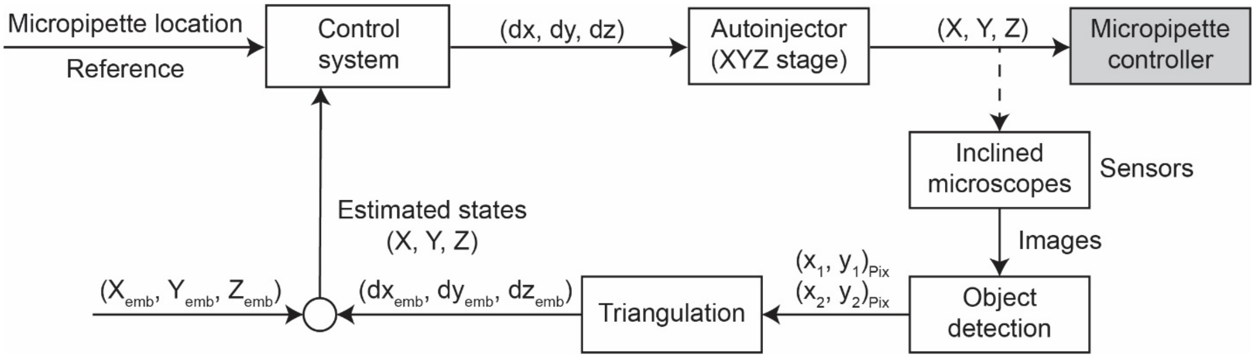

Figure S4: Procedure for microinjection location estimation: In the automated microinjection system, the micropipette remains stationary, serving as a reference point for the control system. The control system output, denoted as dx, dy, dz, is then applied to the XYZ stage of the autoinjector, determining the XYZ location of an embryo. The microinjection point is of primary interest in this context. To detect the microinjection point on an embryo, two inclined microscopes function as sensors in the control loop. These microscopes capture images, which undergo processing through object detection algorithms to identify X and Y pixel locations from each microscope. Utilizing these X and Y pixel locations, the dx, dy, dz in conjunction with the global XYZ embryo locations, are then used to determine the final XYZ locations of the embryos. The control system, using the estimated states and reference locations, generates the next dx, dy, dz output for the XYZ stage. This comprehensive procedure for microinjection point estimation was used in the microinjection of both Drosophila and zebrafish embryos.

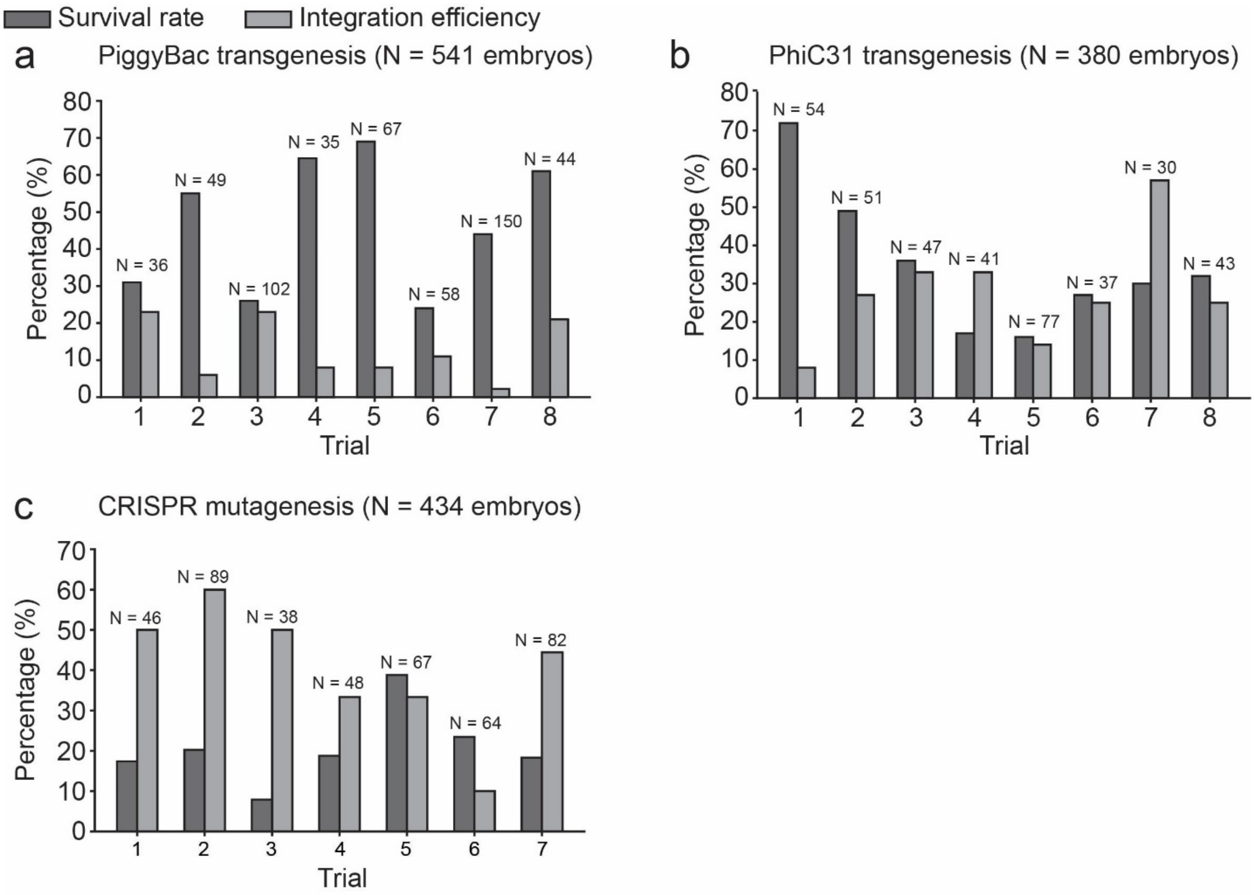

Figure S5: Trial-by trial breakdown of post-injection survival rate in germline transgenesis experiments conducted in Drosophila: (a) PiggyBac transgenesis, (b) PhiC31 transgenesis, and (c) CRISPR mutagenesis. Number of embryos injected (N) are shown on the plots.

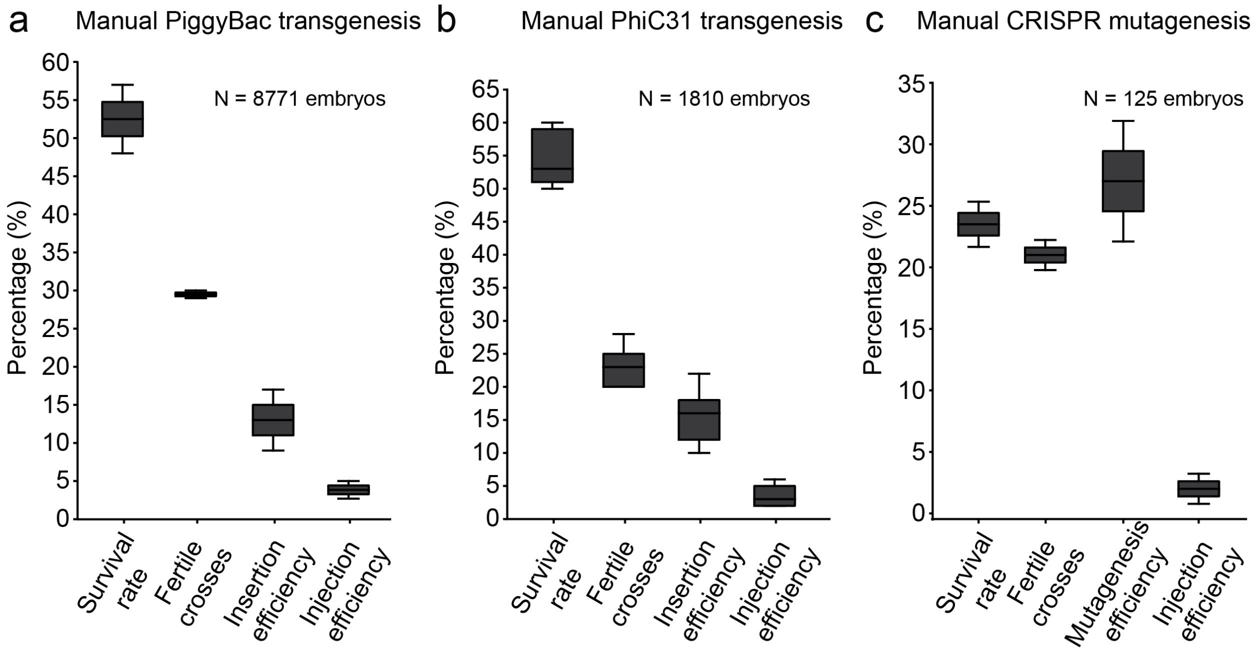

Figure S6: Post-injection survival rates, % fertile crosses, insertion efficiencies and injection efficiencies obtained with manual microinjections in Drosophila (a) PiggyBac transgenesis, (b) PhiC31 transgenesis, and (c) CRISPR mutagenesis.

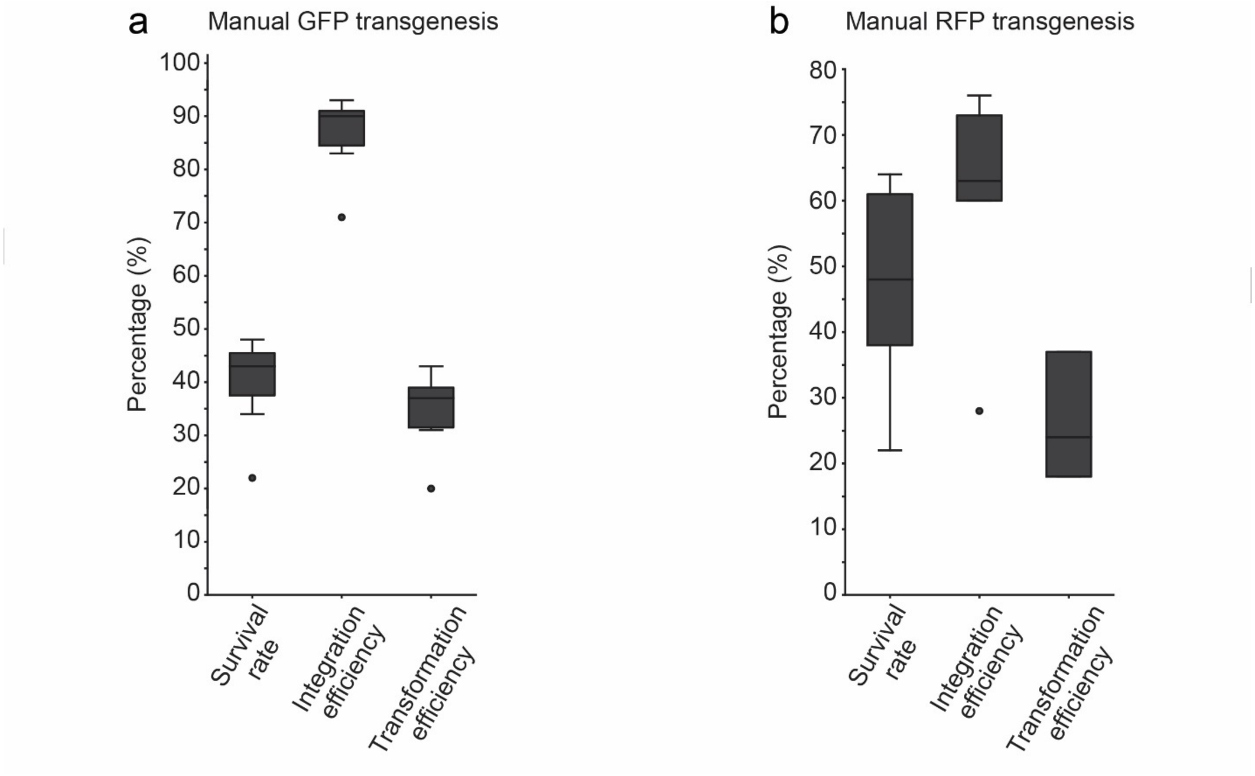

Figure S7: Post-injection survival rates, integration efficiencies and transformation efficiencies obtained with manual microinjections in zebrafish: (a) GFP transgenesis and (b) RFP transgenesis.

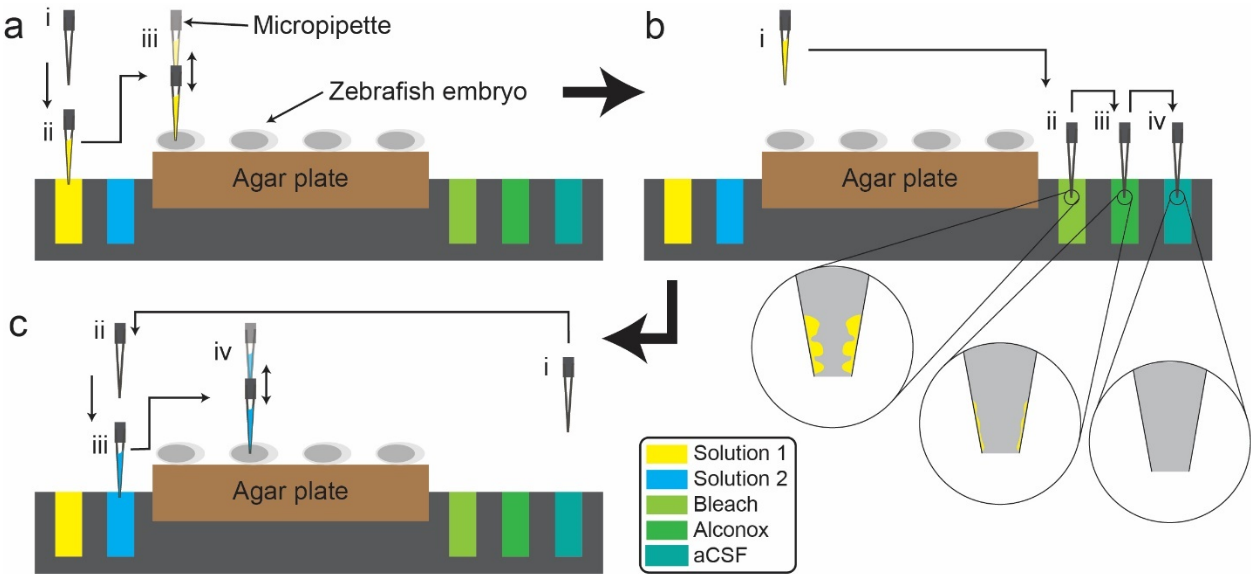

Figure S8: Multi-solution system: Multi-solution system used for zebrafish embryo microinjections. (a) microinjection of an embryo with solution 1: i) Micropipette before micropipette front filling ii) front filling of first solution iii) microinjection of first solution (b) cleaning of micropipette: i) micropipette after microinjection of first solution ii) cleaning of micropipette via bleach, iii) Alconox, and iv) aCSF, and (c) microinjection of an embryo with solution 2: i) micropipette after cleaning ii) micropipette before micropipette front filling iii) front filling of second solution iv) microinjection of second solution.

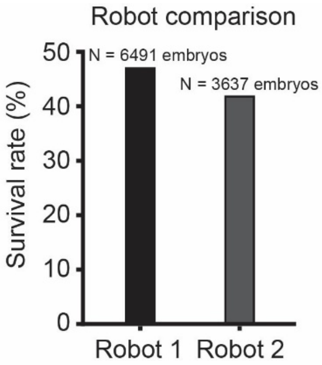

Figure S9: Robot comparison: Larvae survival rate comparison of two independent microinjection robots used for Drosophila embryo microinjections.

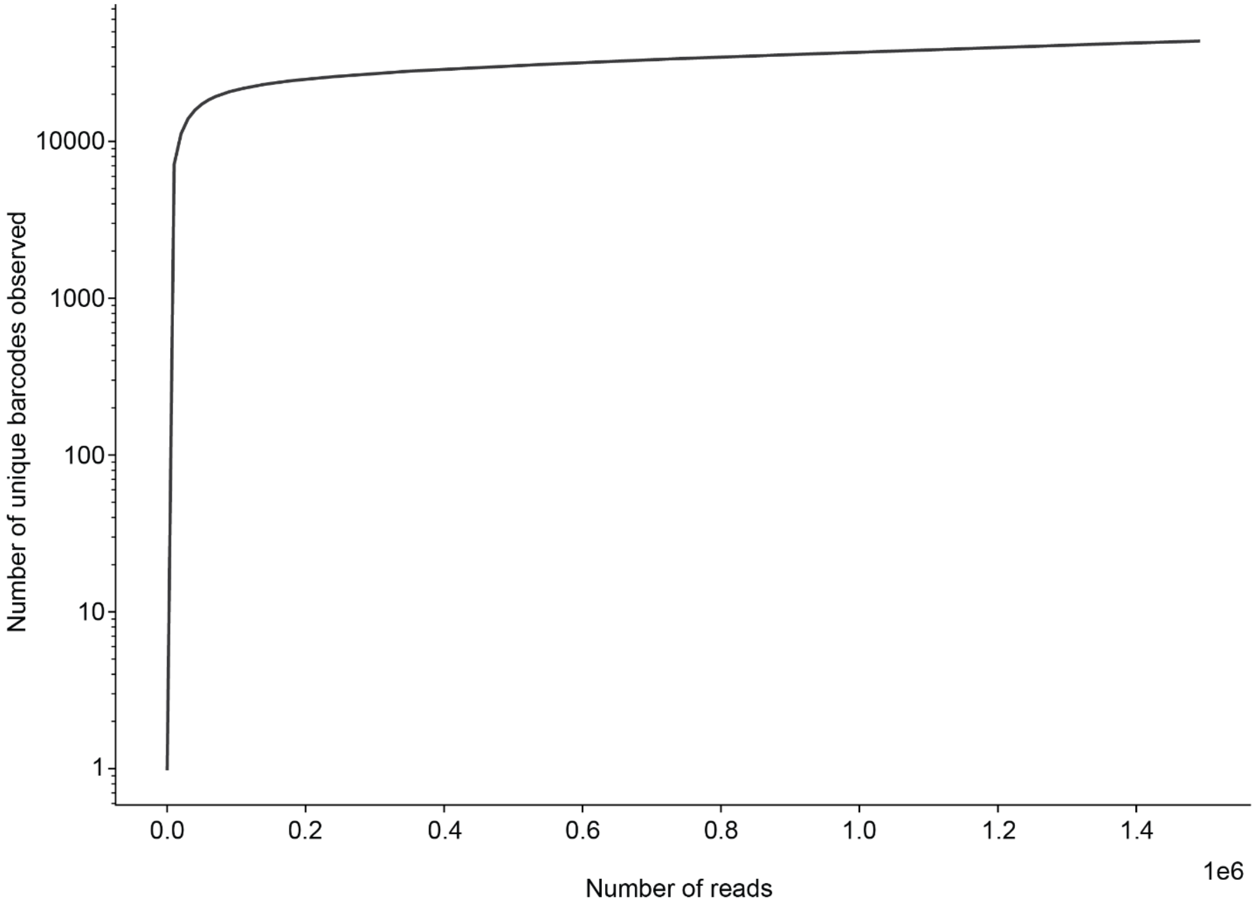

Figure S10: Barcode library saturation: Barcode library saturation plot.

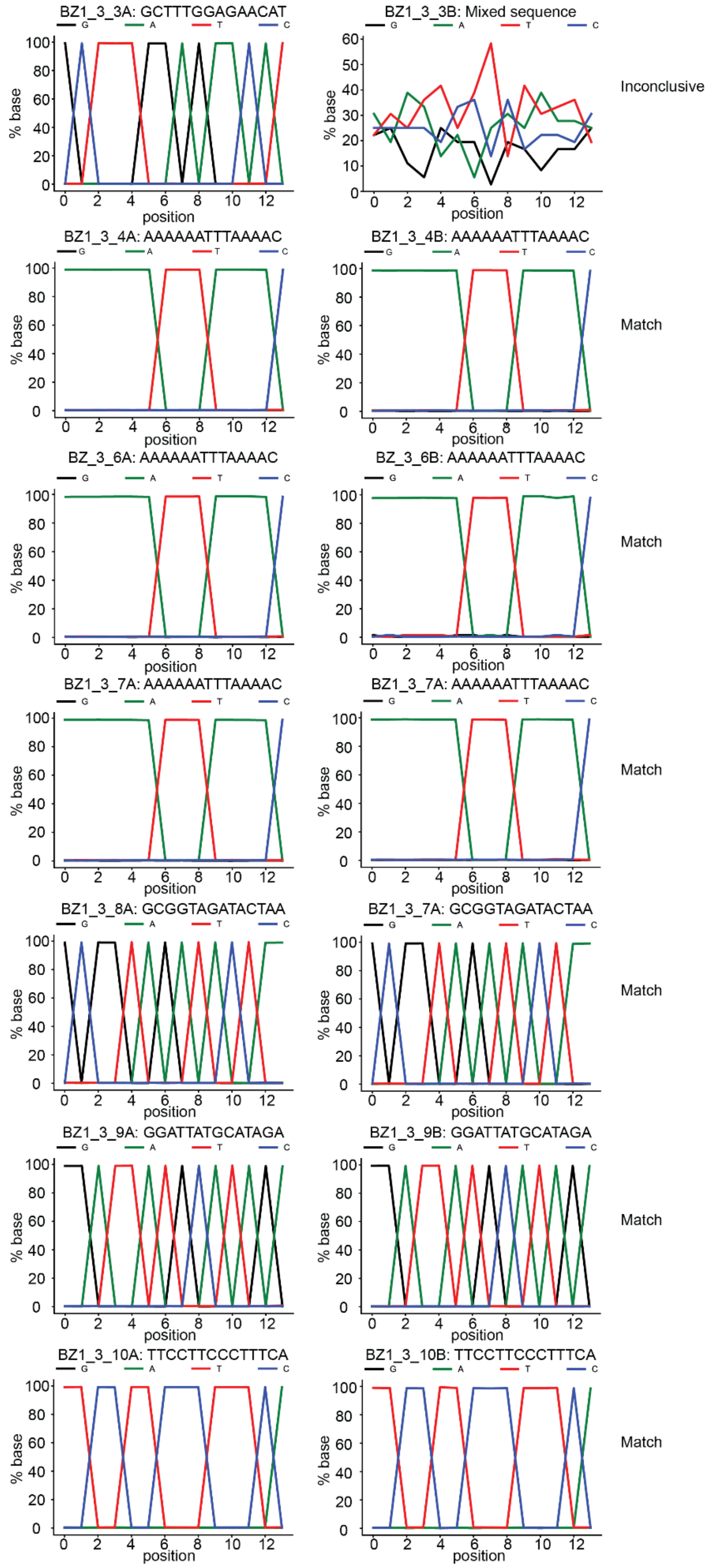

Figure S11: Barcode sequencing comparison: Comparison of replicate barcode lines extracted and sequenced on independent plates.
