# Supplementary material for: High-throughput genetic manipulation of multicellular organisms using a machine-vision guided embryonic microinjection robot
Source: Genetics. 2024 Feb 19;226(4):iyae025. doi: 10.1093/genetics/iyae025 (PMC10990426; doi:10.1093/genetics/iyae025)
Supplement: iyae025_Supplementary_Data [file iyae025_supplementary_data.zip › Table_S1_GENETICS-2023-306540.pdf]

	Description	Source	Quantity
**Microscope**			
	AC254-150-A	Thorlabs	2
	SM1A39	Thorlabs	2
	C6W Cage Cube	Thorlabs	4
	B1C Blank Cover	Thorlabs	8
	SM1CP2	Thorlabs	8
	SM1V05	Thorlabs	3
	SM1L30	Thorlabs	2
	ER6 Cage Rod	Thorlabs	8
	2X Objective	Edmund Optics	2
	SM1A3	Thorlabs	2
**XYZ Stage**			
	BA1	Thorlabs	4
	MSB15/M	Thorlabs	1
	TPA-0348A-00: X	TPA	1
	TPA-0348A-04: X	TPA	1
	Power Supply	Amazon	2
**DSLR Setup**			
	RA90	Thorlabs	4
	RA180	Thorlabs	4
	PH6	Thorlabs	4
	TR12	Thorlabs	2
	TR6	Thorlabs	4
	DSLR Camera	Amazon	1
	DSLR Power cab	Amazon	1
	DSLR USB cable	Amazon	1
	DSLR Holder	Amazon	1

**Micropipette Holder Setup**			
	Micropipettes (AF	Sutter Instrumen	1
	Micropipette holc	Warner Instrume	4
	Micropipette handle		4
	Pressure Regula	Proportion Air	1
	Injection Setup	3-D Printed	1
	Micropipette holc	3-D Printed	1
	AB90	Thorlabs	4
	Power Supply	Amazon	1
	Pressure cord (C	Radwell	1
**Inclined Microscopes Setup**			
	PH6	Thorlabs	4
	PH4	Thorlabs	4
	TR6	Thorlabs	20
	TR4	Thorlabs	4
	TR3	Thorlabs	8
	RA90	Thorlabs	12
	Hayear camera	Amazon	2
	USB Cable	Amazon	2
**Electronics**			
	Arduino Nano	Amazon	1
	LED	Amscope	1
	MicroUSB Cable	Amazon	1
**Thorlabs Miscellaneous Parts**			
	MB2424 - Alumir	Thorlabs	1
	SPW602	Thorlabs	1
	HW-KIT2 - 1/4"-2	Thorlabs	1
	Hex and Alien Ke	Thorlabs	1
